# Enhancing ESL students’ academic achievement in expository essay writing using digital graphic organisers: A mixed-methods research

**DOI:** 10.1016/j.heliyon.2023.e15589

**Published:** 2023-04-20

**Authors:** Godswill U. Chigbu, Ngozi U. Emelogu, Cajetan I. Egbe, Ngozi G. Okoyeukwu, Kingsley O. Eze, Chidinma K. Nwafor, Chioma P. Patrick, Okon E. Okon, Philomina A. Agbo, Frederick A. Okwo

**Affiliations:** a Department of English and Communication, The Hong Kong Polytechnic University, Kowloon, Hong Kong SAR China; b Department of Arts Education, University of Nigeria, Nsukka Nigeria

**Keywords:** Graphic organisers, Digital graphic organisers, English language, Expository essay writing, Metacognitive strategy, Thematic analysis

## Abstract

Students in Nigerian English language classrooms encounter difficulty in writing. However, the utilisation of metacognitive strategies has the potential to aid students in organising their thoughts during writing for better achievement. Therefore, this study aims to examine the effect of digital graphic organisers on secondary school students’ achievement in expository essay writing and the students’ perceptions of writing challenges and the impact of the strategy. The study adopted a mixed-methods research design consisting of a within-group experimental design and focus group interview. Five research questions and one hypothesis are formulated to guide the study. An intact class size of 38 students is the subject of the study, while an expository essay writing achievement test and a focus group interview were used for data collection. Percentage, mean and standard deviation, and thematic analysis were used in answering the research questions, while a paired sample *t*-test was used to test the null hypothesis at 0.05 significance. The study found a statistically significant difference between students' mean achievement scores before and after exposure to digital graphic organiser charts when writing expository essays.

## 1 Introduction

The continuous mass failure in the English language in the senior secondary school certificate examinations conducted by WAEC and NECO has become worrisome. According to the WAEC Chief Examiners' reports [1,2], this failure has always been mainly attributed to poor writing skills. This causal factor is relatable because writing has been described as the most complex and sophisticated language skill [3]. Hence, it is a highly valued literacy skill in the classroom and beyond. Moreover, unfortunately, many learners struggle to write effectively [4], including Nigerian students who encounter difficulty in writing. The teaching and learning of English centres on ensuring that students become competent in the four language skills: listening, speaking, reading, and writing. Among these four language skills in the English language, writing or essay writing occupies a prominent position. Essay writing is an essential skill all students need in their academic lives. It provides an avenue to demonstrate one’s understanding and knowledge of a topic. Through essay writing, students narrate unique experiences and describe people, events, places, and processes. They also develop and defend logical arguments or provide expository information on diverse concepts and issues. Writing an essay involves using cognitive abilities to understand any subject matter [5]. The involvement of cognitive abilities develops students’ higher-order thinking skills.

As former secondary school English language teachers and WAEC examiners, we observed that students need help to compose well-structured essays during class writing exercises and examinations. Their essays are either characterised by vague ideas about the essay topic, incoherence, especially at the sentence and paragraph level or being markedly shorter than the expected number of words, which is clear evidence of a lack of ideas and how to develop them. The non-employment of teaching strategies by most Nigerian teachers may worsen these scenarios. They rely primarily on the traditional method, which is lecturing. However, research has somewhat demonstrated that metacognitive strategies might improve students’ writing abilities. Based on this perspective, the study would examine how the employment of digital graphic organisers, a metacognitive strategy, could impact on senior secondary school students’ academic achievement in an expository essay. Specifically, the study aims to answer the following questions.

1. What percentage of students recorded positive gains after exposure to the digital graphic organisers?
2. What percentage of the students moved from low to higher writing skills due to the use of digital graphic organisers?
3. What is the effect of digital graphic organisers on the mean achievement scores of students in expository essay writing?
4. What are the perceived difficulties students encounter while writing expository essays?
5. What are the perceived effects of using digital graphic organisers in writing expository essays?

### 1.1 Graphic organisers

Graphic organisers have a very significant impact on the English language classroom. Since the introduction of the theory of multiple intelligences by Gardner [[6], [7], [8], [9], [10]], classroom instructors are considering integrating multimodal strategies to facilitate learners’ comprehension. Gardner’s theory of multiple intelligences states that learners assimilate and internalise new information in an instructional context where more than one mode of learning is employed. According to the theory, "students possess different kinds of minds and therefore learn, remember, perform, and understand in different ways" [ [6], p. 11]. This argument advances the essence of multimodality in every aspect of the instructional process, from the instructional content to the mode of delivery and assessment. In the theory of multiple intelligences, Gardner proposed eight types of intelligence: bodily-kinesthetic, logical-mathematical, interpersonal, intrapersonal, musical-rhythmic, naturalist, verbal-linguistic, and visual-spatial intelligence. The graphic organisers characteristically fit into this theory because they are spatial and visual modes of learning [11]. Hence, it engages the visual-spatial intelligence of the learners. Besides, McKnight [11] posited that graphic organisers are essential and effective teaching tools for generating and organising ideas and content and aiding learners' comprehension. Employing graphic organisers for instructional purposes falls under strategy-based instruction. Its essence and effectiveness lie in how it enables students to see practically the relationships, links, and connections between terms, ideas, facts, and information.

Graphic organisers are grounded in metacognition and core cognitive theories such as cognitive constructivism and schema theory. Graphic organisers are metacognitive strategies. Metacognitive strategies enable students to organise, regulate, and evaluate their learning. According to Flavell [12], a metacognitive strategy is an effective strategy that reveals knowledge of cognition processes. Inclusive Schools Network [13] defined metacognitive strategies as "designed methods employed to help students understand the way they learn or make them "think" about their "thinking." There are different types of these strategies, namely regulation checklists, thinking aloud, mnemonic aids, self-questioning, and graphic organisers, to mention only these. A graphic organiser, one of the metacognitive strategies, can be created manually, by drawing or digitally using software [14]. Beyond being a metacognitive strategy, the basis and activities of graphic organisers are anchored in cognitive theories. Scholars of cognitive theories, for instance Refs. [15,16], have proposed that learning occurs based on organised and predictable mental structure and thought processes. Cognitive theories propose that graphic organisers are powerful tools that enable instructors to interact with learners’ schemas. This interaction enhances the learners’ information retrieval and retention. Anderson [15] defined schemata as a mental mode that generously represents an individual’s knowledge of the world and lived experience and serves as the basis for interaction, response, and analysis of similar cognitive experiences. Slavin [17] explained that with schemata, learners could input, store, and retrieve codes or information hierarchically. It also offers the structure for constructing and representing a complex idea and a reason for how experience impacts the acquisition of new knowledge.

Graphic organisers provide templates of what learners know and how they know them. Di Cecco and Gleason [18] noted that GO charts are visual and spatial representations of information that arrange logical relationships between facts, concepts, or ideas. GO charts are employed to help students visualise and organise ideas. Without them, students’ ideas may become cluttered and difficult to capture coherently in a draft. In addition, GO charts allow for the complete utilisation of brain skill regions, aid in overcoming information overload, and allow the collection of knowledge and resources in one location [8].

Furthermore, they boost creativity by allowing people to think more freely and helping them perceive information more thoroughly. Also, they clarify concepts via relationships and structure, which assist humans in problem-solving, decision-making, and action; while enhancing memory and understanding [19]. The good things about this strategy are mainly well established in native speakers’ English classrooms. However, how effective it could be in an ESL classroom needs attention.

Although, these arguments, so far, have favoured the graphic organisers. However, there are also criticisms that contend against graphic organisers in relation to the theory of multiple intelligences because there is no adequate empirical data supporting the claims. The criticisms are that optimal performance is because of the complex skills acquired and developed through nurtured abilities and conscious exercises against any supposed unique and innate abilities [20,21]. Nevertheless, as much as these criticisms exist, a well-documented body of empirical literature [[22], [23], [24]] has demonstrated that graphic organisers facilitate good writing performance, idea generation, and reading comprehension. Different kinds of graphic organisers include flowcharts, Venn diagrams, concept maps, story maps, templates, cognitive maps, and visual displays. Different definitions have been provided for graphic organisers. According to Stull and Mayer [25], a graphic organiser consists of spatial arrangements of words or a group of words intended to represent the conceptual organisation of the text.

### 1.2 *Digital* graphic organiser

Graphic organisers are classified into paper-based and computer-based types [26]. Paper-based graphic organisers are organisers produced using papers and drawings on paper. On the other hand, computer-based organisers may be distinguished into full computer-based graphic organisers and semi-computer-based graphic organisers. While the former refers to computer-based graphic organisers produced as computer applications or software, the latter is about graphic organisers with some of the same flexibility as the full computer-based graphic organisers. This study focuses on the latter, *digitally* created graphic organisers (or DGOs). It is also called a web graphic organiser [27].

Moreover, "*digital*" qualifies graphic organisers in this context in two ways. Firstly, it defines the medium of design and production of these graphic organisers, which are through software such as Microsoft Word, Google Docs, and CorelDraw; specially built web or mobile apps; or even editable templates downloaded from online sources. Secondly, it is *digital* because of the editable and reusable features of the charts when they are created or downloaded through digital platforms. *Digital* graphic organisers allow students to adapt, customise, or personalise their work and reduce paper waste [28]. The word "*digital*" here is italicised to capture that it is not entirely technological. It is not an app or software, but it requires teachers and students to create an organiser chart using Microsoft Word or Google Docs. Moreover, students can practise prewriting and writing using the soft copy at home, in the language skill computer lab, or in an ICT-supported classroom. Teachers and students can also print it out for use in the classroom. The emphasis on *digital* here is on creating graphic organiser charts. This process may give the students the perception that they are managing, controlling, or creating what they learn. For Brown [29], the idea of *digital* in his study was about using a smartboard to project the graphic organisers while the students perform writing tasks in the classroom. This process may appear slim, but it is very significant and shows improvement since, in the Nigerian context, material-based instructional strategies are always improvised using handcrafts involving painting and drawing.

**Fig. 1:**
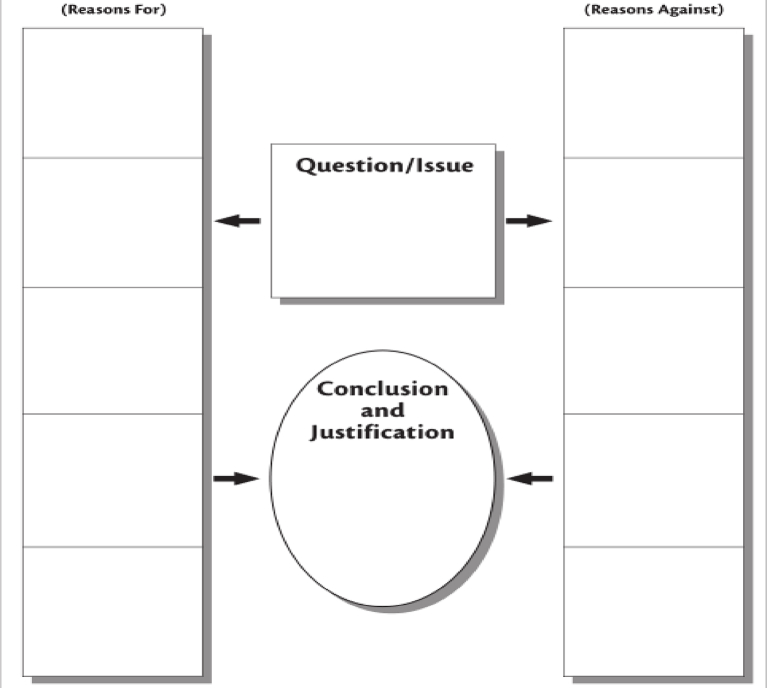
Persuasion chart. Source: authors.

**Fig. 2:**
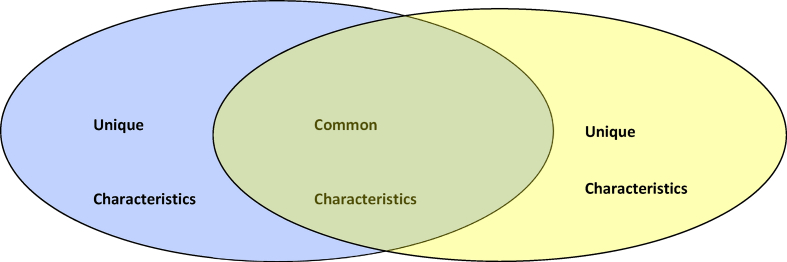
Comparing/contrasting Chart. Source: authors.

**Fig. 3:**
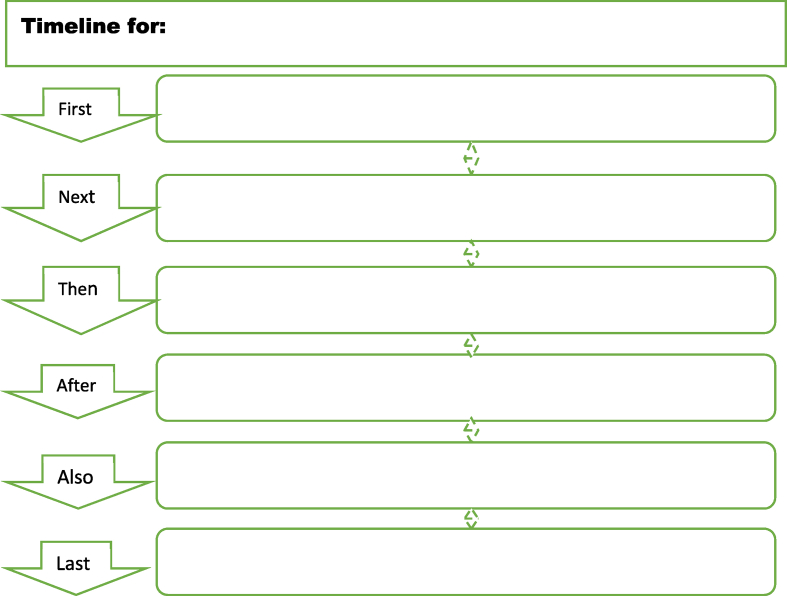
Sequencing chart. Source: authors.

**Fig. 4:**
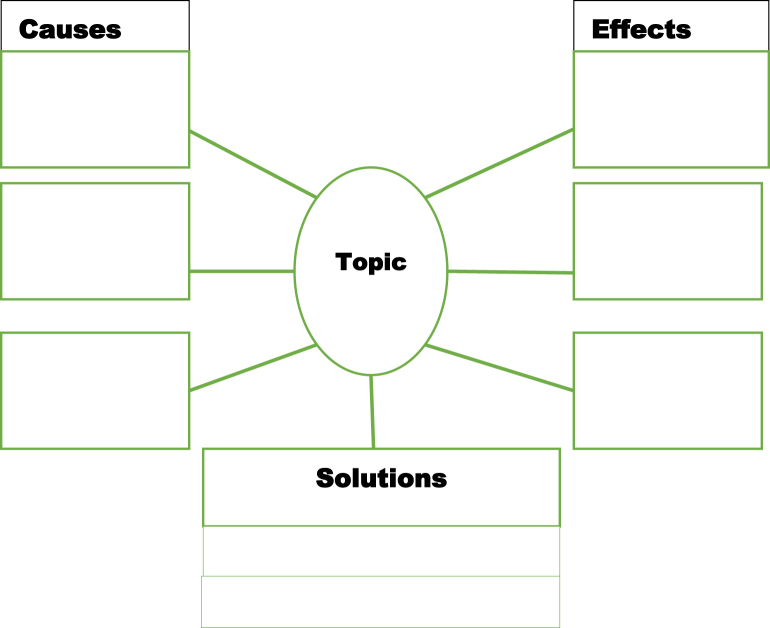
Cause and Effect Chart. Source: authors.

There are different formats or types of digital graphic organisers, including cause-and-effect charts, compare-and-contrast charts, sequencing charts, persuasion charts, etc. (see Fig. 1, Fig. 2, Fig. 3, Fig. 4). The cause-and-effect charts help students visualise the connection between a particular cause and its effect or the interrelationship between a problem and its primary causes, effects, and solutions. The compare-and-contrast organisers visually depict the similarities and differences between ideas or concepts. With this chart, the main ideas can be represented with supporting or opposing ideas. The ideas can further be compared or contrasted against one another. The sequence chart illustrates events. The chart can be used to outline actions from beginning to end. The actions can be represented top-down or left-to-right, connected by arrows. The persuasion chart allows students not only to identify evidence in support of their argument but also to acknowledge opposing opinions. (See Ref. [11] for more samples of graphic organisers for teaching writing and other language skills). The students for this study will be exposed to different chart formats and guided on how to use them to write an excellent expository essay.

### 1.3 Expository essay

Acquiring good writing skills through expository essays is of immense benefit to students. This is because, through expository essay writing, students learn to think critically and write analytically using factual, logical, and statistical evidence. According to The On-Campus Writing Lab & The OWL at Purdue [ [30], Para. 1], "the expository essay is a genre of essay that requires the student to investigate an idea, evaluate evidence, expound on the idea, and set forth an argument concerning that idea clearly and concisely." It is characteristically unbiased and impersonal in an academic tone and with factual information. In addition, the ability to write an expository essay engenders a problem-solving skill because this genre requires students to analyse a topic critically through "comparison and contrast, definition, example, and the analysis of cause and effect" [ [30], Para. 1]. This approach to writing an expository essay aligns with the different formats or types of digital graphic organisers, such as cause-and-effect charts, compare-and-contrast charts, sequencing charts, and persuasion charts detailed above.

The writing skill acquired at the secondary school level enables students to cope with term papers and projects, which form the bulk of academic work at higher institutions. Moreover, at the place of work, expository essay skills enable individuals to produce numerous reports and business proposals. Consequently, Wise [31] remarked that expository essay writing teaches students how to use language to inform and educate others, which are necessary skills for making positive changes in one’s life and the world. Therefore, expository essay writing needs to be constantly taught in an ESL classroom using exciting and practical strategies to enable learners to acquire the required writing skills. This is also because it is one of the genres of writing in which students are assessed in external examinations such as the West Africa Senior School Certificate Examination (WASSCE) and the National Examination Council (NECO).

Students' essay scores are likely to predict their achievement in other subjects. Research [32,33] suggest that students’ ability to write good essays correlates with success in various academic subjects. Research reports on students’ writing in Nigeria have consistently revealed that students are predominantly deficient in essay writing. Teachers are unsatisfied with the quality of the essays students produce [34]. Writing good essays is hard for most students and may be more difficult for students in the ESL classroom.

Consequently, it becomes crucial that every student learns to produce good essays. However, writing essays is challenging and demanding [35]. These students face difficulties in this language skill related to spelling, sentence formation, word choice, capitalisation, and punctuation. They also lack domain-specific vocabulary and writing style [36]. Their difficulties differ from that of Native English students [37]. Furthermore, students struggle with the cognitive processes of planning and organising their ideas and thoughts for the essay topic [38]. These peculiarities are resolvable if metacognitive strategies, such as graphic organisers, could be incorporated into the learning process [39]. In a learning activity such as writing, students should be able to self-regulate the process, use prior knowledge intentionally, and, at best, think about their thoughts.

DGO charts may be essential in expository essay writing. This is because they are “thinking technologies”. In an ESL classroom, student's writing and thinking abilities are examined through essay writing tests. Students who do not have strong writing skills will not be able to pass external examinations (WASSCE and NECO) since Paper 1, which tests writing ability, carries the most points (50%). Narrative, expository, descriptive, debate/argumentative essays, speeches, and articles are among the essay categories that students are assessed on. From the authors’ observations, students struggle to organise ideas when writing essays. For instance, in most narrative essays, the actions may end up not being well sequenced; in their expository essays, the effect or solution to a problem may be omitted. Such problems may be resolved if DGO charts are used as a thinking guide in the writing process.

Furthermore, students are faced with the cognitive demand to analyse a topic critically, and DGO charts enable students to develop the cognitive and metacognitive skills to do the analysis required in the mastery of essay writing skills. Klimova [40] posited that writing enhances students' cognitive and meta-cognitive skills, and the use of visual aids will trigger the critical thinking of students, which will indirectly contribute to their cognitive and metacognitive development. Cognitively, students can think out ideas and analyse and synthesise them. At the same time, with metacognition, they will structure these ideas logically and monitor and evaluate them as they develop them in paragraphs. DGO charts enhance the thinking abilities of students when brainstorming, generating ideas, linking those ideas, and sequencing events. Utilising DGOs may increase concentration on essential ideas.

### 1.4 Review of related literature on the usage of graphic organisers

Some empirical studies have been done using graphic organisers. Studies [[41], [42], [43], [44]] have proposed that graphic organisers affect students’ writing achievement and even performances in disciplines other than language. Odewumi and Gambari [7] investigated the efficacy of graphic organisers on junior secondary school students' cognitive writing development skills. Their research revealed that students taught using graphic organisers outperformed those taught using conventional methods. Kansizoğlu [8], adopting a meta-analytical approach, examined 70 experimental/quasi-experimental studies on the effect of graphic organisers on language learning and instruction conducted between 2000 and 2016. The results obtained from the study were interpreted based on the random effects model. It was found that graphic organisers have a more significant effect on academic achievement than traditional teaching. More so, graphic organisers have made teaching writing skills easier. Finally, Tayib [45] examined how graphic organisers affect students' writing abilities and attitudes regarding this vital language skill. The participants in the study were 24 males who were enrolled in the preparation programme during the academic year 2012–2013 at Umm Alqura University in Saudi Arabia. The participants' writing scores were compared and examined quantitatively before and after the graphic organisers' intervention to discover whether there was any evidence of variations between the mean scores. In addition, the survey results on writing attitudes were qualitatively evaluated to see whether any changes had occurred in students' attitudes. The study's findings suggest that graphic organisers may enhance students' writing abilities and influence their attitudes regarding writing skills.

Some studies have been done on using graphic organisers in prose and narrative writing. For example, Uba et al. [9] assessed the use of graphic organisers in understanding the prose genre, aiming to emphasise their usage in Nigerian classrooms. The study aimed to determine whether secondary school students in Nigeria studying prose literature-in-English and are taught with graphic organisers do better on prose and comprehension tests. Four senior secondary schools with a sample of 100 students were purposefully chosen for the study. The selected schools were divided into graphics-based schools (GBS) and non-graphic-based schools (NGBS). The students of GBS were taught using eight visual organisers, whereas the NGBS was used as a control. The investigation, which was analysed using descriptive statistics and an independent sample *t*-test, disclosed that graphic organisers could allow learners to control the learning process in prose literature classes. It concluded that these organisers might ensure students’ understanding and achievement. As a result, the authors suggested that graphic organisers be used in the classroom instructional processes across subjects in Nigerian secondary schools.

Moreover, Sharrock [46] investigated the effect of a concept map, a graphic organiser, on students’ writing. The six-week study involved one third-grade class that was observed on two occasions. First, they gave a narrative essay test without a graphic organiser, and second, another with graphic organisers. The scores of the two observations were analysed using a paired sample *t*-test. The study revealed that students demonstrated more growth when they utilised the graphic organiser as part of the writing process than when they did without the graphic organiser in the first test.

Research has been done on improving the specifics of writing components using graphic organisers. Styati and Irawati's [10]study showed that utilising graphic organisers improved the students in terms of content, organisation, grammar, and mechanics. Unzueta and Barbett [47] reported that students who used computer graphic organisers to write persuasive essays recorded increased word count and supporting details in the composition. Beyond these studies, also [48,49], have also proven that graphic organisers are effective for organising written discourse. Although several studies attest to this, most only used paper-graphic-organisers. Therefore, the “*digital”* graphic organisers might work differently than paper organisers. With this, it becomes essential to investigate the effect of digital graphic organisers on the expository essay writing process in the Nigerian English language classroom.

### 1.5 Theoretical framework

This study is anchored on "Dual Coding Theory," developed by Paivio [50]; "Schema Theory," introduced by Bartlett [51] and further developed by Anderson [15]; and "Cognitive Load Theory," developed by Sweller [52]. The choice of these theories is informed by the research done by the Institute for the Advancement of Research in Education (IARE) [53], which found that three cognitive learning theories, namely "Dual Coding Theory," "Schema Theory," and "Cognitive Load Theory," support the use of graphic organisers in the learning process. Dual coding theory proposes that humans code information in both verbal and nonverbal ways. According to schema theory, schemas or information networks are inside the memory. Learners can use graphic organisers to link new material to the current knowledge stored in the schemas.

Conversely, cognitive load theory accepts that working memory (short-term memory) has a maximum capacity to handle information; thus, when the load is surpassed, learning does not occur. When used correctly, DGO charts minimise cognitive strain and allow access to additional resources, allowing new content to be learned [53]. Similarly, Ellis [54] argued that visual organisers minimise the need for significant information processing abilities necessary to acquire a subject, make the information much more comprehensible by organising the information content, and allow the material to be handled at more sophisticated levels. Therefore, it is left to be seen how DGO charts will affect students' achievement in expository essays.

### 1.6 Hypothesis

Ho1 there is no significant difference in the mean achievement scores of students after and before their exposure to digital graphic organisers.

## 2 Research method

### 2.1 Design

The study adopted a mixed-methods research design. According to Creswell and Creswell [ [55], p. 59], “Mixed methods is an approach to research in which the investigator collects, analyses, and interprets both quantitative and qualitative data (close-ended information), integrates or combines the two approaches in various ways, and frames the study within a specific type of design or procedure”. The design was found appropriate for this study because it allowed for the triangulation of the findings. The study adopted a within-group experimental design, particularly a pre-test-post-test design. Salkind [56] defined this design as a research design where two or more measures are obtained from a sample of subjects. The study used a single case study design (SCD) based on the qualitative approach because the study aims to explain the perceptions of senior secondary school students toward the use of digital graphic organisers and to evaluate any difficulties in the students' use of graphic organisers. Bogdan and Bilken [57] defined a case study as "a detailed examination of one setting, or a single subject, a single repository of documents, or one particular event." Five questions and one hypothesis were formulated to guide the study.

### 2.2 Participants of the study

The study participants were one class of 38 SS2 students at the University of Nigeria Secondary School, Nsukka. The 38 students were exposed to graphic organisers and were administered essay tests pre- and post-exposure. Furthermore, the study employed a focus group for the case study. This focus group was sampled from the entire class using a purposive sampling technique. The study adopted a focus group because interviewing all the class members would be impossible or challenging. Therefore, purposive sampling was used to sample seven (7) students based on gender and academic achievement. Using the teachers’ records, the seven students were four male students and three female students. Two of the seven students were high achievers, another two were average achievers, and the last three were achievers. This sampling of these participants based on gender and academic achievement characteristics was done not to study their differences or similarities but to create an aggregate or all-encompassing view of the students. Also, seven students were selected because, according to Johnson and Christensen [58], between six and twelve people are ideal participants for a focus group interview (FGI).

### 2.3 Ethical consideration

The Research Ethics Committee of the Faculty of Education at the University of Nigeria, Nsukka, approved this study. The authors also strictly adhered to the American Psychological Association's (2017) [59]ethical standard for conducting research with human participants. We obtained consent from the participants, the intact class form teacher, and the school management.

### 2.4 The instrument for data collection

The data collection instruments were the Expository Essay Writing Achievement Test (EEWAT) and focus group interview. Two essay tests were adopted from the 2014 and 2015 West African Senior School Certificate Examination (Paper I) expository essay tests for Nigerian students. One was used as a pre-test, and the other was administered as a post-test. An equivalent form reliability index of 0.714 was obtained for the test. The instrument measured the student’s performance before and after using DGO charts. The test was scored following the WASSCE analytical method of grading essays: content = 10; organisation = 10; expression = 15; and mechanical accuracy = 5, a total of 40. A score of 20 (50%) of the total mark is the benchmark. A score of less than 20 indicates that the learner has lower writing skills, while a score of 20 and above indicates that the learner has higher writing skills. The total number of learners that scored less than 20 was determined. The scores of these learners in the post-test were compared with their scores in the pre-test after being exposed to DGO charts. The number of students that migrated from a lower writing skill to a higher writing skill and the percentage, given the sample, was determined. This number indicated the percentage of those with lower writing skills that migrated to higher writing skills.

The interview was deemed appropriate for this case study design because "interviews are an essential source of case study evidence because most case studies are about human affairs or actions. Well-informed interviewees can provide important insights into such affairs or actions. The interviewees can also provide shortcuts to the history of such situations, helping you identify other relevant sources of evidence" [ [60], p. 121]. The interview was an open-ended focus group interview (FGI). For Krueger and Casey [ [61], p. 11], a focus group interview offers "a more natural environment than that of an individual interview because participants are influencing and influenced by others—just as they are in real life".

### 2.5 Procedure and method of analysis

**Fig. 5:**
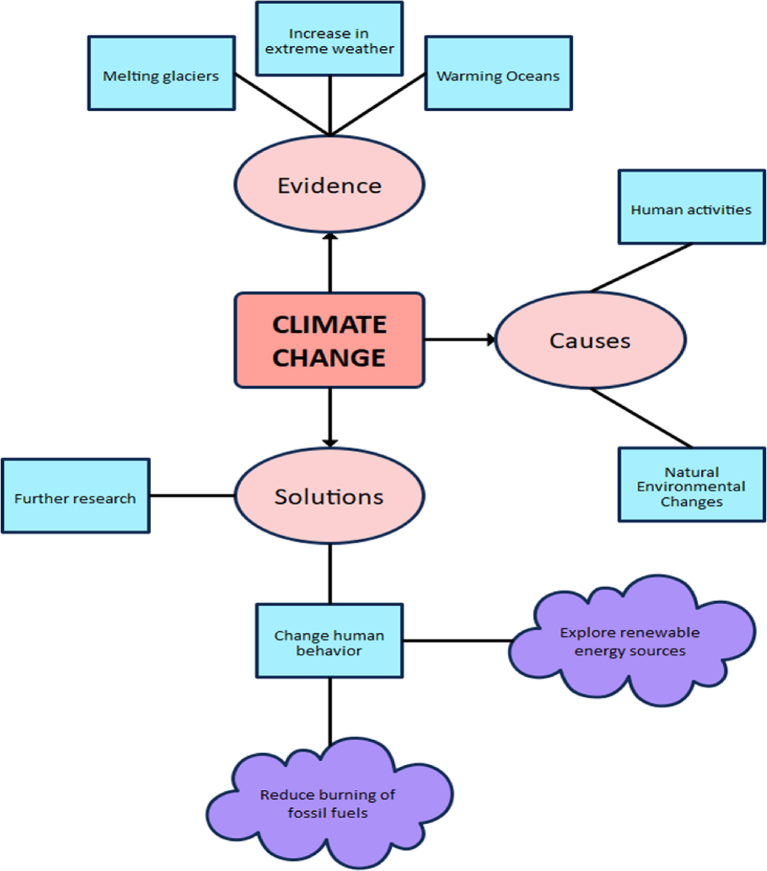
Cause and effect organiser on climate change. Source: EdrawMax Template Community.

The research lasted for five weeks. A phase one test (i.e., a pre-test) was administered to the students on the first week. The second, third, and fourth weeks introduced students to the four DGO charts illustrated in Fig. 1, Fig. 2, Fig. 3, Fig. 4, Fig. 5 and how to use them to organise ideas, thoughts, and points when writing expository essays. Fig. 5 is one of the DGOs used for classroom practice to teach the students during the exposure. More so, during the three weeks of exposure, editable sample templates of these graphic organisers were sent to the email addresses provided by the students. They were to download them, follow the pattern and create their own, adapt the charts to their preferred style, or use them just as they are to practise. In phase two (i.e., post-test), another test was administered to the students on the first day of the fifth week, while the remaining four days of the week were used to assess the essays. During the test, the students came with clean sheet printouts of their designed or downloaded charts and used them in the essay writing test's prewriting, writing, and revising stages. In the prewriting stage, the students brainstormed on the essay topic and then organised and outlined their points using the charts. The DGO charts helped the students to think about the ideas discussed in the essay and how they would organise them. When writing their essays, they constantly referred to the DGO charts. At the revising stage of the writing, which includes editing and proofreading, the students evaluated their essays with the DGO charts to ensure they were in tandem. The students followed the diagram of the chart to develop their points. They typed in ideas about causes in the “causes” squared boxes, ideas about effects in the “effects” squared boxes, and ideas they could think of about solutions in the “solution” rectangular boxes (Fig. 4 is an example of the chart they used). The percentage was used to answer the first and second research questions, mean and standard deviation were used to answer the third research question, and FGI was used to answer the fourth and fifth research questions. A paired sample *t*-test was used to determine whether the mean score of the phase one test was not significantly different from the mean score of the phase two test at a probability level of 0.05.

The study used FGI for research questions 4 and 5 to explore students’ perceptions regarding the challenges they encounter while writing expository essays and the advantage and disadvantage of their exposure to the DGOs. The FGI is chosen because we need to directly learn students’ difficulties in writing an expository essay and how they felt regarding gaining or not gaining from their exposure to the use of DGO. Besides, FGI ensures a considerably high level of participant involvement in spontaneous responses from students. The students interact with the interviewer and one another, which can offer more accessibility to the students' perception [62].

With the assistance and permission of the class teacher, the focus group were assembled in a convenient venue within the school premises that was devoid of noise or distraction. Two of the researchers conducted the interview. The researchers created a conducive environment by employing humour to make the group feel at ease. The FGI was conducted in two sessions. The first session was used to answer the RQ4 and was held before the treatment exposure; the second session answered the RQ5 and was conducted after the treatment exposure. During the FGI, in addition to audio recording, one of the students clerked the meeting. The researchers also jotted down their observations during the interview in a journal. After the FGI had been conducted, the audio recording was transcribed. The transcribed data were analysed using thematic analysis. Thematic analysis is defined as "a method for systematically identifying, organising, and offering insight into patterns of meaning (themes) across a data set" [ [63], p. 57]. We followed the 6-phase procedure for conducting a Thematic Analysis [64,65]. In inserting extracts of the transcribed interview in the discussion, the focus group members are identified using the pseudonyms hyphenated with subscript letters _m_ or _f_ to indicate a male or female member.

## 3 Results

1. What percentage of students recorded positive gains after exposure to the digital graphic organisers?

**Fig. 6:**
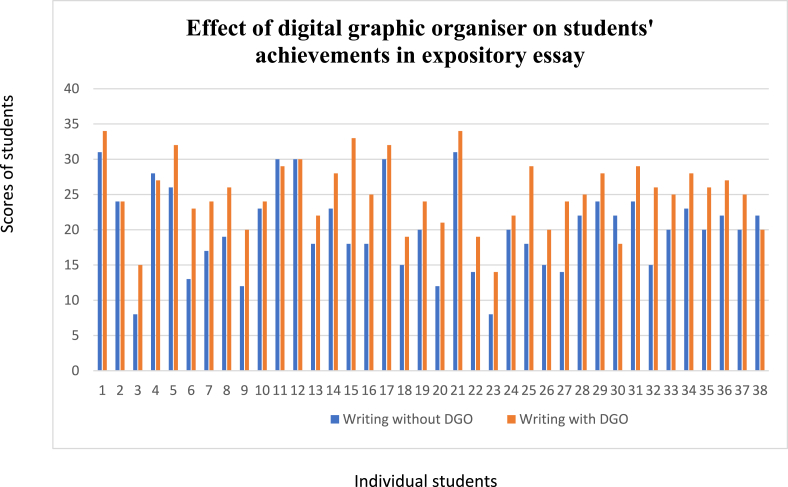
Students' migration from low to higher writing skills due to the use of digital graphic organisers.

According to Fig. 6, 84.21% of participants recorded positive gains in the phase two essay test after their exposure to DGOs; 10.53% maintained equal performance in the phase one and two essay tests even after exposure to DGOs; therefore, there was no positive gain. On the other hand, 5.26% demonstrated negative gain as their scores in the phase two essay test after exposure to DGOs were less than in the phase one essay test.

2. What percentage of the students moved from low to higher writing skills due to using digital graphic organisers?

31.58% of participants with lower writing skills moved to higher writing skills after exposure to DGOs.

3. What is the effect of digital graphic organisers on the mean achievement scores of students on expository essay writing?

Data in Table 1 reveals that before being exposed to digital graphic organisers for expository essay writing, students had a mean pre-test score of 20.23 and a standard deviation of 6.08. However, after being exposed to digital graphic organisers, they had a mean post-test score of 25.02 and a standard deviation of 4.91. Therefore, it shows that exposure to digital graphic organisers considerably affects students' achievement in expository essay writing.

**Table 1:** Mean ($\bar{X}$) and standard deviation (SD), t value of achievement scores of students pre and post exposure to digital graphic organisers.

Intervention	N	$\bar{X}$	SD	t	df	Sig (2-tailed)
Pre exposure (Pre-test)	38	20.23	6.08	7.547	37	.000
Post exposure (Post-test)	38	25.02	4.91			

The data in Table 1 also shows the paired *t*-test analysis of the significant difference in the mean achievement scores of students after and before their exposure to digital graphic organisers in expository essay writing. The table reveals that *t* (37) = 7.547, p < .000. The null hypothesis is rejected since the probability figure (sig.2-tailed) of 0.000 is less than 0.05. Therefore, there is a statistically significant difference between the mean achievement scores of students after and before being exposed to digital graphic organisers. The students performed better after they were exposed to digital graphic organisers.

### 3.1 thematic analysis

Thematic analysis was adopted to address research questions 4 and 5. With the thematic analysis, codes were generated, and common themes were stated. For Research Question 4, three themes were generated: critical essay blockage, dis-preference to expository essays, and lack of organisational skill. Table 2 below presents the themes and their codes. The themes are essential for defining the participants’ perceptions. Of course, certain parts of the participants’ perceptions overlap across themes. However, this should be taken as an interpretation of participants’ perceptions, and each theme is not mutually exclusive. Table 3presents the themes and codes of the perceived effects or gains of using digital graphic organisers in writing expository essay.

4. What are the perceived difficulties students encounter while writing expository essays?

**Table 2:** Theme and codes of the perceived difficulties students encounter while writing expository essays.

Theme 1: Critical essay blockage	Theme 2: The Dis-preference for expository essay	Theme 3: Lack of organisational skill
• Lack of ideas • Unable to generate enough ideas • Regurgitating ideas • Difficulty in developing ideas • Lack of ideas • Lack of word choice • Poor vocabulary • Short word count	• Expository essay topics are about current affairs • Impersonal topics • Expository essays are factual, not imaginative • Lack of interest in current affairs • Expository essay topic requires factual ideas • Preference to imaginative writing • Students should be drilled on expository writing by the teachers • The expository essay topics are unfamiliar	• difficult organising ideas • Lacks organisational skills • No knowledge of the conclusion part • Difficult segmenting ideas

**Table 3:** Themes and codes of perceived effects of using the digital graphic organisers in writing expository essays.

*Theme 4:* Digital Graphic Organiser aids in the Organisation of Ideas and Thoughts	*Theme 5:* Benefits of using a Digital Graphic Organiser in Writing
• Digital Graphic organiser organises paragraphs • Digital Graphic organiser helps to generate ideas • Digital Graphic organiser helps to organise thoughts • With digital graphic organisers, ideas are clear enough • It helps to segment ideas • It helps to group my ideas	• Digital Graphic organiser is a guide • Digital Graphic organiser is a like a google map • Digital Graphic organiser helps to reach the word count • With digital graphic organiser, there is no anxiety while writing • Following the digital graphic organiser my ideas are developed seamlessly • Digital Graphic organiser helps explore all aspect of the topic • Sticking to digital graphic organiser makes the essay lengthy • Digital Graphic organiser helps to visualise • Always refer to the digital graphic organisers while writing • Digital Graphic organiser helps to create mental pictures of thoughts • Producing Digital graphic organiser involves the students in the instructional process • Digital graphic organiser helps me to take charge of my learning and writing

### 3.2 Critical essay blockage

This theme is defined as the difficulty or inability to generate and develop new and original ideas, a lack of topic-specific vocabulary, and a short word count when writing an expository essay. The repetitive mention of the codes “lack of ideas” and “unable to generate enough ideas” points to how taxing it is to come up with new ideas. The codes “difficulty in developing ideas” and “regurgitating ideas” reveals participants struggle with developing ideas. The codes “lack of word choice” and “poor vocabulary” denote how constrained the participant’s ability is in effectively expressing themselves. The concept of “critical” in this theme emphasises two things: the formality nature of the expository essay and the critical stage of the participants, which is the stage of learning.

### 3.3 Dis-preference for expository essay

This theme is defined as students’ attitudes and beliefs towards writing that centre on their lack of awareness and interest in current affairs, which majorly are sources of topics for expository essays. The codes also highlight what students prefer with respect to the topic. This lack of awareness of current affairs makes expository topics unfamiliar. The dis-preference is occasioned too by the fact that teachers do not drill students on expository.

### 3.4 Lack of organisational skill

We defined this theme as the challenges in organising ideas when writing an expository essay. These challenges include learners’ difficulty structuring an essay to capture every aspect of the topic at content and structural levels. For example, at the content level, it is the inability to discuss causes and effects/solutions or to compare ideas in an expository essay. At the structural level, this theme means being unable to form a good introduction and lacking the skill to develop paragraphs seamlessly. It is also the inability to form a good conclusion for an expository essay. All of these, summarily, indicate the challenges in effectively planning and organising thoughts.

For Research Question 5, two themes were generated, and they are: digital graphic organiser aids in the organisation of ideas and thoughts and benefits of using a digital graphic organiser in writing. Table 2 below presents the themes and their codes.

5. What are the perceived effects of using the digital graphic organisers in writing expository essays?

### 3.5 Digital graphic organiser aids in the organisation of ideas and thoughts

The definition of this theme is that using digital graphic organiser helps generate, organise, and synthesise ideas and thoughts, both in terms of their content and the structure of an expository essay. The codes suggest that digital graphic organisers help organise paragraphs, generate ideas, and organise thoughts. The use of digital graphic organisers makes understanding the relationships between different ideas and concepts easier to understand.

### 3.6 Benefits of using a digital graphic organiser in writing

The codes point to the various benefits of using a digital graphic organiser in writing, such as self-regulation, guidance, ease of navigation, help in reaching the word count, reducing anxiety, seamless idea development, exploring all aspects of a topic, and promoting lengthiness in an essay. The codes demonstrate that the production of digital graphic organisers involves the students in the instructional process and allows them to take charge of their learning.

### 3.7 Discussion of the findings

The findings of the study indicated that, generally, students’ performance in essay writing improved with their exposure to DGO charts. The study majorly established a significant difference between students' mean achievement scores before and after exposure to DGO charts in expository essay writing. The students recorded a significant gain in achievement after appropriating the metacognitive strategy in writing the expository test. This indicates that the improvement is necessitated by exposure to DGO charts. This is informed by the fact that the DGO charts enabled the participants to think out ideas concerning the topic of the essay and have their main and supporting ideas well outlined and organised. Instead of racking their brains during writing, the students used the DGO charts to think about their thoughts in the prewriting stage. The DGO charts served as reference guides for them during the writing and revising stages.

Out of the four charts exposed to the students, they mostly used the causes and effects and sequencing charts. Hence, 90% of the phase two essays indicated that the students organised and sequenced the contents or thoughts of essays in the following way: causes, followed by effects, and lastly, solution. The arrangement of these thoughts made their essays have good content and organisation. The essays were also written in good expression, that is, the grammar. Errors pertaining to mechanical accuracy were reduced significantly in the phase two test compared to the phase one test. The students' use of digital graphic organisers improved the writing components of their expository essay's content, organisation, expression (grammar) and mechanical accuracy. This finding agrees with Styati and Irawati [10], whose study found that appropriating graphic organisers in writing improved students’ writing the content, organisation, grammar, and mechanics. When assessing essays, the higher the word count, the higher the score assigned to each writing component.

The 84.21% students who recorded positive gain in their achievement have essay scripts with higher word count in the phase two than in the phase one. This means that planning one’s writing in the prewriting stage using digital graphic organisers does not only facilitate generating ideas, but organising, and drafting them extensively. This submission corroborates the findings of the study by Unzueta and Barbett [47]. In general, the findings of this study agree with [7,41,[44], [45], [46]] that graphic organisers have a great effect on students’ writing achievement. Beyond the statistical data indicating positive and negative gains on the students’ exposure to the DGOs, a focus group of the participants expressed the difficulties they encounter when writing expository. The reason for appropriating FGI to understudy these difficulties is that it allows the group members to be personal with their opinions.

With regards to research question 4, one of the difficulties students encounter in essay writing is critical essay blockage. Critical essay blockage is related to creative blockage. A creative blockage is a well-known phenomenon that has been linked to burnout, anxiety, and stress in the field of psychology. It may also be caused by external factors such as time constraints, lack of materials and poor guidance. In Mihaly Csikszentmihalyi’s Flow Theory of Creativity [66], the state of flow and optimal experience is necessary for creative processes. However, the creative blockage could occur in the creative process when a task taken up is too high or low compared to a person’s skill level, leading to a loss of motivation and creativity.

I find it difficult to write an expository essay because it doesn’t give me room to forge ideas. I prefer imagining things and writing about them. But expository essay topics require factual information. (Ifeoma-_f_)

Critical essay blockage, a difficulty students encounter, may arise from a lack of interest, time-constraint, inadequate resources, and poor guidance from the teachers. Also, Critical writing blockage is grounded in Albert Bandura’s theory of self-efficacy, which postulates that people’s beliefs about their abilities to accomplish a task impact their motivation and performance. In other words, believing that one lacks the ability, as the participant stated, could lead to critical writing blockage.

Another difficulty is that they have a dis-preference for expository essays. Dis-preference to expository essay is grounded on a few theoretical principles such as social cognitive theory and self-determination theory. Social cognitive theory suggests that the prior experiences and beliefs of learners and the views of those around them can shape and influence their attitude towards writing an expository essay. More so, motivation plays a huge role in students’ attitudes towards learning-this is the standpoint of Self-determination theory. This theory suggests that students are more motivated to learn when they perceive the learning content as meaningful and relevant.

Expository essay topics are always drab and boring. I feel our parents should be the ones bothering themselves about current affairs and not us, the younger ones (Emeka-_m_)

The above statement expresses a lack of motivation and interest which stems from the fact that he does not consider the topics of expository essay that come from current affairs relevant to his generation. This implies that teachers should aim to make the expository essay more meaningful and relevant to the students’ lives by tailoring the topics to suit their interests and incorporating contemporary materials in the English language classroom. Voke [67] suggested that current affairs can be used to teach English, but not by “reading dry and difficult news articles or holding a controversial political debate”. It involves leading the students to find out about the news through English newspapers and current affairs magazines, browsing news using smartphones and engaging them in photojournalism.

The final difficulty the students encounter is a lack of organisational skills. Expository writing as a type of essay demands clear and organised thoughts in well-developed and structured paragraphs. In addition, an expository essay must have a captivating introduction, a well-developed body, and a strong conclusion. These features can be challenging for students struggling with organising their thoughts and forming conclusions, which leads to difficulties in writing good expository essays.

The difficulty I experience all time is how to organise my ideas. I actually know what to write. I have the points I want to explain in my mind. But arranging the ideas so that they flow well becomes impossible. (Obinna_-m_)

According to the self-regulation theory, writing is a complex process that requires goal-setting, self-monitoring, and self-evaluation [68]. A digital graphic organiser is an effective strategy and tool to self-monitor and self-evaluate oneself while writing an expository essay. Effective organisation and planning skills play a vital role in these processes and can impact the success of writing outcomes. Individuals who struggle with organising ideas and forming conclusions in expository writing may face difficulties with self-regulation and may benefit from digital graphic organisers to enhance their organisational and planning abilities.

With regards to research question 5, students perceive the digital graphic Organiser as an aid in the Organisation of Ideas and Thoughts. This positive attitude and perception give credence to the theory that visual aids such as graphic organisers can aid in the cognitive process of organising and synthesising information. Digital graphic organisers align firmly with visual learning theories, such as the Dual Coding Theory proposed by Paivio [50], which suggest that visual aids such as graphic organisers can enhance the process of organising and comprehending information. Additionally, authors such as Novak and Gowin [69] have developed the concept of “knowledge maps” as a tool for organising and generating ideas.

Another perception of the students overlaps with the former on the benefits of using a digital graphic organiser in writing. This suggests that a digital graphic organiser can serve as an effective tool for writers to structure and visually organise their ideas, thus leading to improved writing quality. The codes also indicate that a digital graphic organiser can help reduce anxiety while writing, enabling writers to better express their ideas and reach their desired word count.

For me, viewing those charts while writing, I felt like I was being guided by a google map. It was more than helpful. I knew “what and what” should be in each paragraph by merely looking at the charts. While preparing my own chart, I also learnt how many points and paragraphs I will write (Chinwe-_f_)

Additionally, Chinwe’s statement above highlights the benefit of a graphic organiser in guiding writers to explore all aspects of their topic, thereby promoting lengthiness and depth in their writing. These attested benefits by participants corroborate with research that has shown that digital graphic organisers can support the writing process by helping students to structure their ideas, plan their writing, and generate text [11]. Furthermore, digital graphic organisers have been linked to enhanced writing outcomes such as improved organisation, increased clarity, and more developed arguments [43]. The students

What I enjoyed more was designing my own chart. I did not know I could do it, but I did it. Designing the chart made me know my chart, the boxes and circles very well. (Ejike_-m_).

Furthermore, some of the students, just like Ejike, created their own charts, and the process helped them walk through their essays in their minds. It engaged the students as most of them testified. We found that nearly half of the students created their own chart. It motivated them to participate in the class activities because they perceived the production process of the digital graphic organisers as being involved in the instructional process and, more importantly, taking charge of it. This implies that students’ participation in the learning process could benefit from them being involved in the creation of instructional resources. The finding corroborates with Uba et al. [9] submission that students' use of graphic organisers gave them control of the writing process.

This interview with the focus group has aided in understanding the perceived effects of using digital graphic organisers in writing expository essays. The students considered using DGOs to write expository essays very helpful as the DGOs served as thinking maps. The students were able to brainstorm and arrange ideas using the DGOs templates. Overall, their standpoint on the exposure indicates a shift in perception of how difficult expository essays are to how the students were able to improve on writing expository essays due to the use of DGOs. The DGOs aided the students significantly in the area of metacognition. They gained "thinking awareness" while writing. This achievement is important because "when learners become more aware of their thinking processes, they move beyond simple assignment completion into acquiring and maintaining *a sense of purpose in their work"* (emphasis ours) [ [70], p. 6]. "A sense of purpose in their work" is what Lan [ [71], p. 109] calls "expected outcomes". These are also the "positive gains" we mentioned in this study. Exposing the students to DGOs and allowing them to use them in writing expository essays agrees with Evensen’s [70] view that a "multi-strategic approach that allows students to habitually monitor their own thinking as they complete learning tasks" should be used.

Although the study is diagnostic in nature, its within-group design in the mixed method could be improved upon by further studies by adopting other designs. This limitation does not invalidate the generalisability of the significant difference due to DGOs. However, we recommend that further studies be carried out in Nigeria with different participants and in different areas of study because the use of metacognitive strategies such as DGOs is relatively novel and low in the English language classroom in Nigeria.

The study has practical significance for teachers, curriculum planners, textbook writers, and researchers. The findings of this study bring to the attention of teachers, curriculum planners, and textbook writers the importance of *digital* graphic organisers and how they can be incorporated into classroom activities Teachers can recreate the organisers for their students, while curriculum planners and textbook writers should emphasise the need for using DGOs in their planning and writing. For the researcher, the study would serve as a reference background for their future studies.

## 4 Conclusion

The study aimed to examine the impact of metacognitive strategy, particularly the use of digital graphic organisers, on students' academic achievement in expository essay writing. The study also looked at the perceived difficulties and effects of using DGOs. The findings of the study revealed that students had different challenges concerning writing an expository essay. However, after their exposure, their perception concerning writing an expository essay shifted from the negative to the positive. In fact, many of the participants benefitted greatly from their exposure to DGOs, and a reasonable percentage migrated from low writing skills to higher skills in writing expository essays. Some participants benefited very little, while a few participants did not benefit at all. This might have been due to certain individual variables which should be identified and factored into future research. Generally, the use of DGOs significantly improved students’ achievement in writing expository essays.
